# Occurrence Rates of Delirium in Brain Tumor Patients: A Systematic Review and Meta-Analysis

**DOI:** 10.3390/cancers17121998

**Published:** 2025-06-15

**Authors:** Zachary Tentor, Alexander Finnemore, Paul J. Miller, Joshua Davis, Erika Juarez Martinez, Charlotta Lindvall, Eyal Y. Kimchi, John Y. Rhee

**Affiliations:** 1 Department of Supportive Oncology, Dana-Farber Cancer Institute, Boston, MA 02215, USA; zachary_tentor@dfci.harvard.edu (Z.T.); joshua_davis@dfci.harvard.edu (J.D.); charlotta_lindvall@dfci.harvard.edu (C.L.); 2 School of Medicine, University of Navarra, 31009 Pamplona, Spain; alexanderfinnemore@gmail.com; 3 Department of Neurology, Northwestern University, Chicago, IL 60611, USA; erika.juarez@northwestern.edu (E.J.M.);; 4 Department of Neurology, Harvard Medical School, Boston, MA 02115, USA; 5 Center for Neuro-Oncology, Department of Medical Oncology, Dana-Farber Cancer Institute, Boston, MA 02215, USA

**Keywords:** delirium, brain tumors, glioma, brain metastases, systematic review, meta-analysis, neurological complications, neuro-oncology

## Abstract

**Background**: The occurrence (incidence or prevalence) of delirium in brain tumors is unknown, yet delirium is associated with increased morbidity and mortality and worse quality of life. We conducted a systematic review and meta-analysis to determine the occurrence of delirium in hospitalized patients with brain tumors. **Methods**: PubMed, Scopus, and Web of Science were systematically searched for papers from 1 January 1999 to 12 July 2024, including references from texts. Cross-sectional, prospective, and other cohort study designs were included, and individual case reports, case series, editorials, and reviews were excluded. The included papers were scored using a validated sensitivity analysis tool and tested for quality and bias using funnel plots and Egger’s test. We used random effects models for the summary estimates. We performed subgroup analyses by tumor type, tumor location, delirium subtype, and length of stay. **Results**: Of the 452 studies screened, 27 were included, representing 35,958 patients. The overall occurrence of delirium was 0.17 (95% CI [0.11–0.24]). Delirium occurrence in patients with low-grade gliomas, high-grade gliomas, and brain metastases was 0.10 [0.06–0.16], 0.21 [0.10–0.40], and 0.31 [0.16–0.50], respectively. Compared to the occipital lobe, there was a higher occurrence of delirium for tumors in the frontal (RR 3.08 [1.35–8.22]) and temporal lobes (RR 2.88 [1.22–7.93]). The patients were more likely to have hypoactive (RR 1.61 [1.30; 1.98]) than hyperactive delirium. Delirium was associated with 4.62 additional hospitalized days compared to those without delirium (CI [3.23–6.01]). **Discussion**: We confirmed high occurrence rates of delirium in patients hospitalized with brain tumors. Patients with brain metastases had a higher occurrence of delirium compared to patients with gliomas, and delirium occurrence rates were higher in patients with frontotemporal tumors. Delirium occurrence rates in the literature are very heterogeneous and point toward a need for tailored assessments in patients with brain tumors.

## Simple Summary

Delirium is a sudden state of confusion that can occur in hospitalized patients, leading to longer hospital stays, worse outcomes, and reduced quality of life. While delirium has been well studied in patients with other brain conditions like stroke and dementia, little is known about how often it occurs in patients with brain tumors. Brain tumor patients face unique challenges because their tumors can quickly change brain pressure and disrupt normal brain function, potentially making them more likely to develop confusion. This study examined research from the past 25 years to determine how often delirium occurs in hospitalized brain tumor patients. We found that 17% of all brain tumor patients experience delirium. Notably, the rates were higher in patients with secondary brain tumors (31%) compared to those with primary brain tumors. Among the primary tumors, high-grade glioma patients experienced delirium at a rate of 21%, while low-grade glioma patients had a rate of 10%. Patients with tumors in the front and side areas of the brain were more likely to develop delirium, and those who did develop delirium stayed in the hospital nearly 5 days longer. These findings help doctors better understand which brain tumor patients are at the highest risk for confusion and can guide efforts to prevent and treat delirium in this vulnerable population.

## 1. Introduction

Understanding delirium is critical in cancer care, as this acute confusional state is associated with high morbidity and mortality. It is characterized by an acute onset or fluctuating course, inattention, and either disorganized thought or an altered level of consciousness [1]. Hospitalized patients with delirium have prolonged hospital stays and a significant functional decline compared to those without delirium [2]. Often, delirium is associated with worse mental health outcomes and lower cognitive functioning [3,4,5] as well as worse quality of life (QOL), with impacts in both psychological and social spheres [6,7,8].

Delirium occurrence has previously been studied in large cohorts or systematic reviews for patients with varied neurologic diseases, including acute stroke [9,10], dementia [11,12], and other neurodegenerative disorders [11,13,14]. These studies have yielded robust estimates confirming the risks that patients with these neurologic disorders have for developing delirium [15]. Patients with cancer also experience high rates of delirium, and delirium rates among patients with advanced cancer may be as high as 42% at hospital admission [16,17].

Patients with brain tumors present unique challenges for delirium risk assessments. Unlike patients with slowly progressive neurodegenerative diseases, brain tumor patients have space-occupying lesions that can rapidly alter intracranial pressure, disrupt normal neural networks, and cause focal neurological deficits. The presence of an expanding mass lesion, combined with associated cerebral edema and potential for sudden clinical deterioration, creates a distinct pathophysiological environment that may predispose patients to acute confusional states. Therefore, patients with brain tumors may experience higher rates of in-hospital delirium compared to other neurological populations. However, patients with brain tumors have only been studied in smaller cohorts, with the occurrence of delirium in patients with brain tumors varying widely, from 0% [18,19] to 55% [20]. Methodological factors, such as time periods over which delirium was measured, types of assessments used [21], or the heterogeneity of a patient population, such as in postoperative, general hospitalized, or hospice settings [22,23], may account for some of this heterogeneity. However, there has not yet been a systematic review and/or meta-analysis to determine robust estimates of the overall rate of delirium among this patient population.

Better knowledge of the occurrence of delirium in the brain tumor population is crucial for clinical practice in recognizing which patients are at a high risk for delirium, developing interventions to prevent or treat delirium, improving patients’ QOL, and understanding the pathophysiology of delirium in this patient population. Delirium rates may also vary among tumor types and locations, and a better understanding of how occurrence varies based on tumor type (primary versus non-primary brain tumors) and tumor location may also help target preventative interventions for those at the highest risk [16]. Therefore, the goal of this systematic review and meta-analysis was to estimate the occurrence (combination of incidence and prevalence) of delirium in the hospitalized brain tumor population. Our secondary aims were to look at how brain tumor location affected the risk of delirium, whether delirium affected the hospital length of stay, and classify different subtypes of delirium.

## 2. Methods

### 2.1. Search Strategy

The electronic database search used a sensitive search strategy, employing validated search filters for delirium and brain tumors developed with the help of a librarian from the University of Navarra in Spain (Supplementary S3). The electronic databases that were used included PubMed, Scopus, and Web of Science, which were systematically searched for all papers from the past 25 years, from 1 January 1999 to 12 July 2024, to ensure the reported delirium occurrence rates reflected the more recent standard of treatment. Additionally, references from cited texts within the full-text studies were also searched. Only articles in English, Spanish, and Chinese were included.

### 2.2. Study Population

**Figure 1:**
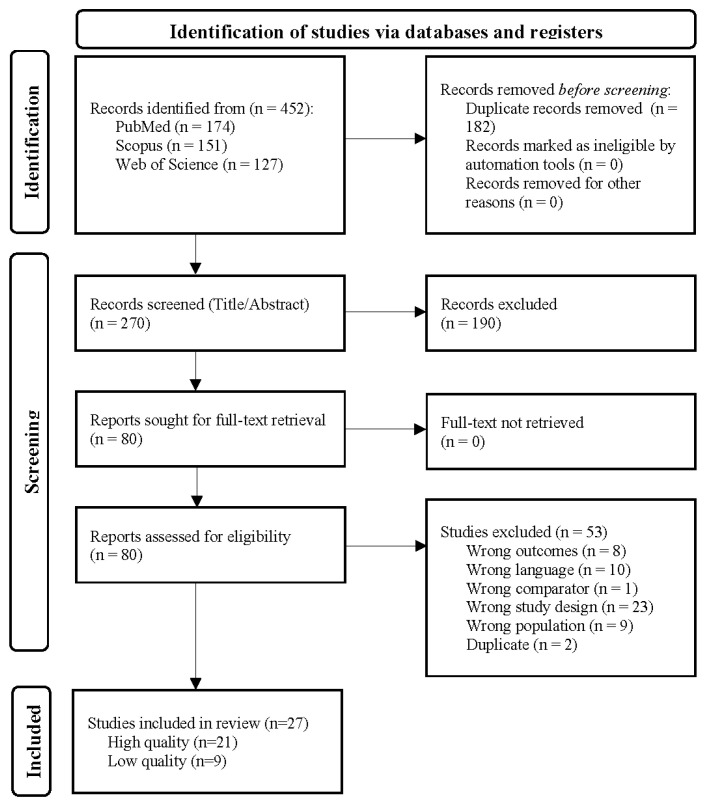
PRISMA flowchart illustrating the selection process for studies included in the systematic review and meta-analysis. The flowchart details the number of records identified through PubMed, Scopus, and the Web of Science, as well as the number of records screened, the number of full-text articles assessed for eligibility, and the number of studies included in the final qualitative and quantitative synthesis.

Studies containing data about the occurrence rates of delirium in patients with either primary or non-primary brain tumors were included. Studies containing delirium data for oncological patients without brain involvement, pediatric populations, or studies not reporting on delirium were excluded. Cross-sectional, prospective, and other cohort study designs were eligible. All prospective studies reported cumulative incidence. We excluded individual case reports, case series, editorials, and reviews (Figure 1).

### 2.3. Data Collection and Extraction

Two authors first independently reviewed each article for inclusion based on the titles and abstracts, followed by full-text reviews, data extraction, and quality assessments using the Covidence© platform (https://www.covidence.org). Covidence© is a free, web-based tool used to manage references and data from systematic reviews and allows for a reviewer’s decision to be blinded until consensus is required. Discrepancies were discussed using prespecified inclusion and exclusion criteria, and an agreement was reached, when necessary, with a third reviewer. The systematic review and meta-analyses were completed in accordance with the updated Preferred Reporting Items for Systematic Reviews and Meta-Analyses (PRISMA) 2020 guidelines and checklists which can be found in Tables S1 and S2 [24]. The PROSPERO registration number is CRD42024565359.

The extracted data included the number of patients, delirium and non-delirium counts, setting, postoperative status, mean age, sex, delirium assessment method, length of stay (LOS), notes relating to interventions or study-specific conditions, delirium occurrence, delirium subtype, tumor type, and tumor location. The method of identification for tumor location varied across studies, with some studies classifying multifocal tumors as present in one or more lobes. Data were extracted to Microsoft Excel by two authors separately and then cross-checked to ensure accuracy. The extracted data were then confirmed by a board-certified neurologist for accuracy.

### 2.4. Quality Assessment

The methodological quality was assessed using a modified version of the JBI Manual for Evidence Synthesis [25]. The questions were categorically scored as “yes” or “no”, and, following a consensus review, the categorical ratings were converted to numerical scores (0 = no, 1 = yes). Scores were assigned to questions grouped into the following domains: (1) Were the criteria for inclusion in the sample clearly defined?; (2) Were the study subjects and the setting described in detail?; (3) Was the exposure (brain tumor) measured in a valid and reliable way?; (4) Were objective, standard criteria used for classification of the condition (brain tumor)?; (5) Were strategies to deal with confounding factors stated?; (6) Were the outcomes (delirium) measured in a valid and reliable way?; (7) Was an appropriate statistical analysis used?; and (8) Was the dropout rate discussed (attrition bias)? A full version of this analysis can be found in Supplementary S1.

Each paper was graded separately by two authors (A.F., Z.T.), and when there was a disagreement, a discussion was held with a third author (J.R.) to achieve consensus. The maximum available score was 7. Only papers with a summed score of five or greater were considered to be of high quality. However, all studies were included in the final analysis because in a sub-analysis by high- versus low-quality studies, there was no significant difference.

### 2.5. Data Analysis

The “meta” package for R 4.4.2 was used to make forest plots and determine the occurrence of delirium across all studies using random effects models. Subgroups of glioma and brain metastases patients were analyzed using the “meta” package. Additional subgroups of brain tumor types were unable to be analyzed due to a few studies. A Bayesian network meta-analysis (NMA) using the “BUGSnet” package compared the incidence of delirium between various tumor locations. We used a random effects model with “Frontal” serving as the reference location. The significance levels were set at *p* = 0.05. We used a similar Bayesian NMA to compare the different types of delirium, with “Mixed” as our reference. We did not correct for multiple comparisons, given it is not necessary in Bayesian NMA [26]. We displayed the results graphically using the “ggplot” package in R 4.4.2. To determine the difference in hospital length of stay between patients with or without delirium, a random effects meta-analysis was conducted using the “metagen” function from the “meta” package in R 4.4.2. When not given in the papers, standard errors were approximated.

### 2.6. Data Availability

This review was registered on the PROSPERO International Registry of Systematic Reviews (registration number: CRD42024565359). Data not published within this article will be made available by request from qualified investigators.

## 3. Results

The literature search yielded 452 citations, of which 27 papers and 28 cohorts met the prespecified criteria. Papers were excluded during the full-text review due to the study design not meeting the inclusion criteria. A risk of bias and study quality assessments (Supplementary S2) showed the quality of the included papers, representing a total of 35,958 patients. Of the 27 papers, 18 were retrospective studies [18,19,27,28,29,30,31,32,33,34,35,36,37,38,39,40,41,42] and 9 were prospective studies [43,44,45,46,47,48,49,50,51]. The study quality assessment showed that 6 of the 27 papers were of low quality [27,36,38,41,45,50]. The average age of the included patients was 53.3 years, and the average percentage of women in the studies was 51.0%. The most common measurement for delirium was the Confusion Assessment Method Intensive Care Unit (CAM-ICU) assessment (*n* = 11 studies), followed by clinical judgment (*n* = 8 studies), with other methods (*n* = 8 studies) including the DSM-IV/V, ICD-10 codes, Delirium Rating Scale-R-98 (DRS-98), Nursing Delirium Screening Scale (Nu-DESC), 4 A’s Test (4AT), and the Delirium Observation Screening Scale (DOS) (Table 1). Quality and risk of bias assessments for each paper are presented in Supplementary S2. Stratification by study quality (Supplementary S4) or assessment tool (Supplementary S5) did not sufficiently explain the heterogeneity of the data.

### 3.1. Overall Occurrence

**Figure 2:**
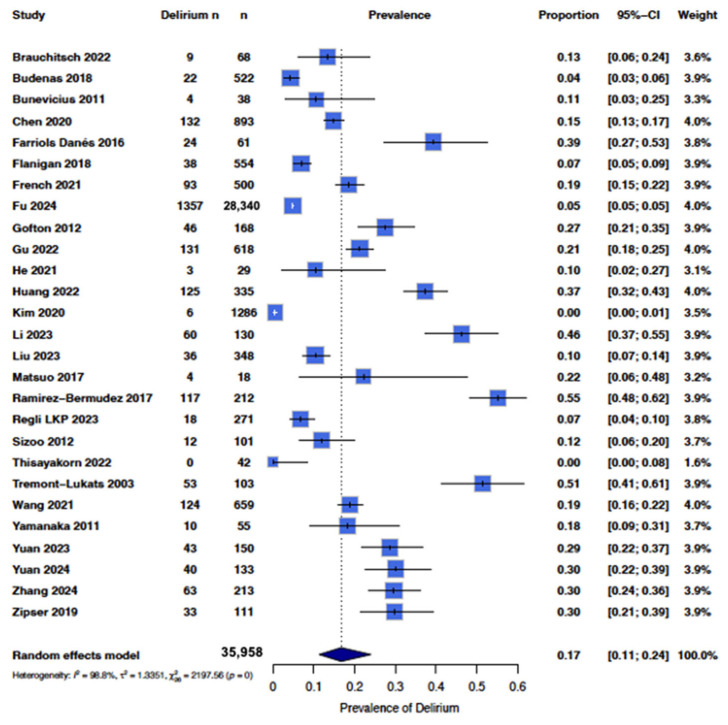
Forest plot showing the occurrence of delirium in all studies. A random effects model in R was used, with the box representing the proportion and horizontal lines representing the 95% CI [18,19,20,27,28,29,30,31,32,33,34,35,37,38,39,40,41,42,43,44,45,46,47,48,49,50,51].

From the 27 papers identified through the literature search and quality analysis, the overall pooled occurrence of delirium in patients with any brain tumor, i.e., all primary and non-primary brain tumors, in our study was 0.17 (95% CI [0.11; 0.24]) (Figure 2).

**Figure 3:**
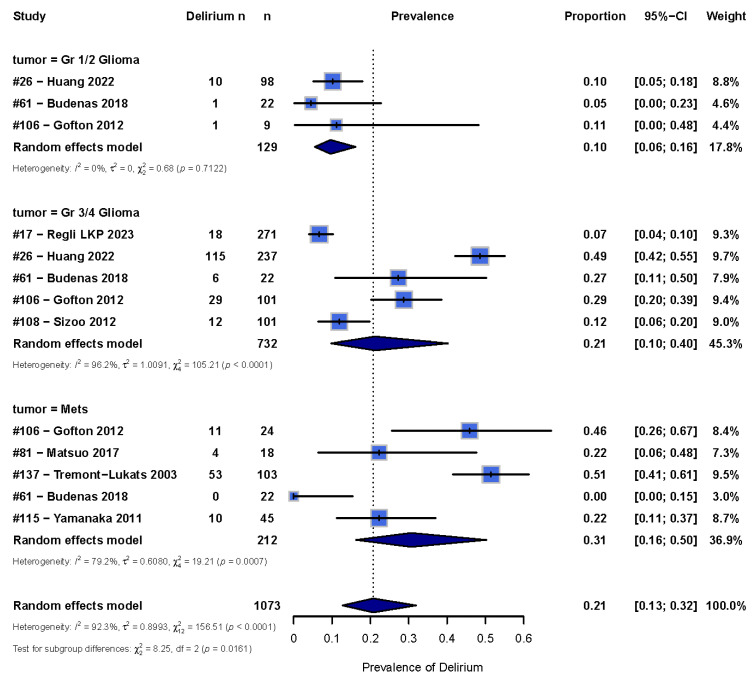
Forest plot showing the occurrence of delirium in glioma and metastases patients. A random effects model in R was used, with the box representing the proportion of delirium and the line representing the 95% CI [33,37,38,39,41,43,44,48].

There was high heterogeneity of the data (I^2^ = 99%), with some studies reporting up to a 50% [29,39] occurrence while others reported less than 10% occurrence [18,19] (Figure 2). Therefore, we sub-categorized delirium occurrence by tumor type where there were sufficient studies to do so. In order to maintain sufficient power, we only included tumor types with at least three studies, which included groups with low-grade gliomas, high-grade gliomas, and brain metastases (Figure 3). The overall pooled occurrence of delirium in patients with glioma or brain metastases was 0.21 (95% CI [0.13; 0.32]). Patients with high-grade gliomas had higher rates of delirium (0.21 [0.11; 0.40]) compared to those with low-grade gliomas (0.10 [0.06; 0.16]). Patients with brain metastases had the highest rate of delirium at 0.31 [0.16; 0.50] compared to high- and low-grade gliomas. A chi-squared analysis showed that occurrence rates were statistically significantly different among the three groups (χ^2^ = 8.25, *p* = 0.02).

### 3.2. Tumor Location

When comparing the effect of tumor location on delirium risk (*n* = 5 cohorts, 490 patients), all studies that were included were from postoperative hospitalized populations [28,30,34,43,50]. These studies reported data on four lobes of the brain—frontal, parietal, temporal, and occipital [52]. Furthermore, two studies reported data on the insular and limbic regions, but these were excluded given the low sample size.

**Figure 4:**
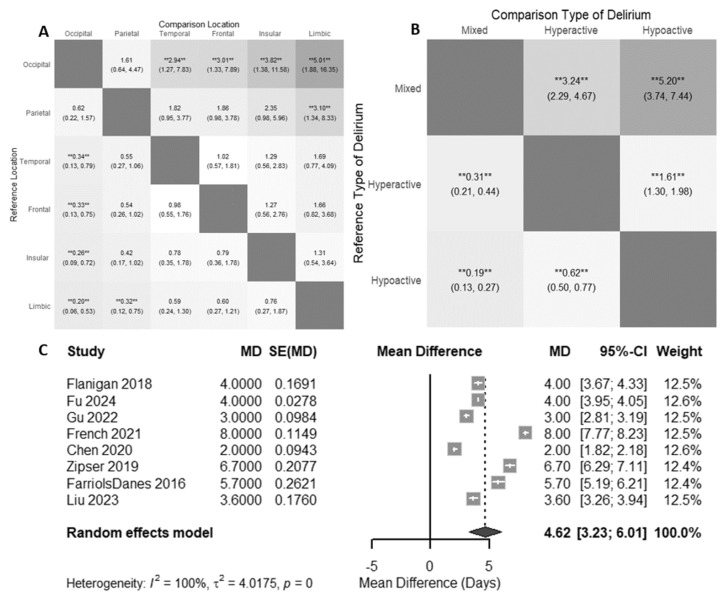
(**A**) Relative risk of delirium by tumor location. Darker squares indicate stronger absolute relative risk. (**B**) Relative risks of delirium subtypes. Darker squares indicate stronger absolute relative risk. The results of the random effects meta-analysis are displayed as relative risks alongside 95% confidence intervals in each cell, demonstrating a comparison between two groups as indicated. ** = *p* < 0.01. (**C**) Mean difference in postoperative length of stay for patients with delirium. When not given in the data, standard errors were approximated [28,29,30,31,32,34,35,51].

The highest risk of delirium (when the reference group was occipital lobe tumors) was for tumors in the temporal lobe (RR 2.94 [1.27; 7.84]) and frontal lobe (RR 3.01 [1.33; 7.89], Figure 4A). Patients with parietal tumors did not have a statistically significantly increased risk for delirium compared to patients with occipital tumors (RR 1.62 [0.61; 4.41]).

### 3.3. Delirium Subtype

From the studies included in our review, a few reported the occurrence of hyperactive, hypoactive, and mixed delirium (*n* = 4 cohorts, 355 patients) [28,30,43,47]. We found that brain tumor patients with delirium were more likely to have hypoactive delirium (RR of 5.20, 95% CI [3.74; 7.44], Figure 4B) or hyperactive delirium (RR 3.24 [2.29; 4.67], Figure 4B) instead of mixed presentations. Additionally, brain tumor patients with delirium are more likely to experience hypoactive delirium than hyperactive delirium (RR 1.61 [1.30; 1.98], Figure 4B).

### 3.4. Length of Stay

When comparing the length of hospitalization for brain tumor patients with delirium and without delirium, using a random effects model, patients with delirium had an additional 4.62 hospitalized days (95% CI [3.23; 6.01], Figure 4C) compared to patients without delirium. Of note, all studies included in the length of stay analysis were looking at delirium in the postoperative context.

### 3.5. Timepoints and Timeframe

In a subgroup analysis for a period of assessment, the occurrence of delirium in studies measuring the occurrence of delirium for a timeframe of equal to or greater than a week was 0.18 (*n* = 10 studies; *n* = 1585 patients, 95% CI [0.09; 0.33], Supplementary S6). For studies measuring delirium with a timeframe of less than a week, the overall occurrence was 0.22 (*n* = 12 studies, *n* = 3600 patients, 95% CI [0.15; 0.30], Supplementary S6). In a subgroup analysis for number of timepoints taken, the studies measuring delirium at multiple timepoints had an occurrence of delirium of 0.21 (*n* = 21 studies; *n* = 4292 patients, 95% CI [0.15; 0.29], Supplementary S7). For studies measuring delirium at a single timepoint, the occurrence was 0.15 (*n* = 1 study; *n* = 893 patients, 95% CI [0.13; 0.17], Supplementary S7).

### 3.6. Publication Bias Assessment

In order to test for publication bias, we analyzed the studies using funnel plots and Egger’s test. For all brain tumors, the Egger’s test resulted in a t-statistic of 3.55 (df = 25; *p* = 0.0016) and a bias estimate of 7.37 (SE = 2.08). This implies that there was statistically significant asymmetry in the data, likely due to both small-study effects and heterogeneity in the data. All brain tumors included craniopharyngiomas, gliomas, and non-primary brain tumors, making the data itself very heterogeneous. A trim and fill test added ten studies (k = 37), which showed, with a random effects model, the new pooled occurrence of delirium to be 0.08 (95% CI [0.04; 0.13]) after adjusting for publication bias. The Q-statistic (4185.20, df = 36) showed heterogeneity (*p* < 0.0001). Funnel plots are shown in Supplementary S8.

Given this, we ran the funnel test again for our subgroup of gliomas and brain metastases, with the Egger’s test for bias showing a t-statistic of −2.07 (df = 11, *p* = 0.0623), indicating that there was no strong evidence of small-study effects or publication bias (Supplementary S8 shows funnel plots). With a trim and fill statistic, given six additional studies were added (k = 19), which showed, with a random effects model, the new pooled occurrence of delirium to be 0.39 [0.22; 0.59] after adjusting for publication bias. The Q-statistic, however, was still 363.24 (df = 18, *p* < 0.0001), indicating that there was still high heterogeneity.

An Egger’s test was unable to be conducted for only gliomas because at least 10 studies are required to run the Egger’s test. However, the funnel plot in Supplementary S8E of just gliomas shows an improved fit compared to all brain tumors as well as gliomas and brain metastases.

## 4. Discussion

We found an overall delirium occurrence of 17% in hospitalized patients with brain tumors. Patients with brain metastases had the highest occurrence rates of delirium at 31%, followed by high-grade glioma patients at 21% and low-grade glioma patients at 10%. Delirium has consistently been shown in the literature to be associated with worse QOL. In order to develop interventions in this patient population to decrease rates of delirium and, thereby, improve QOL, it is important to first understand the scope of the problem, and our findings indicate that a significant proportion of hospitalized brain tumor patients suffer from delirium.

We found that patients with brain metastases were more likely to have delirium than those with low-grade glioma. This could be due to increased neuroinflammation in patients with brain metastases due to the cellular invasion and proliferation in the perivascular or parenchymal space, combined with systemic effects of the primary malignancy and associated treatments that may collectively lower the threshold for delirium development [53,54,55,56]. This is opposed to glioma, where the cancer cells arise from the supportive cells of the brain itself. The fact that patients with high-grade gliomas have a higher risk for delirium compared to patients with low-grade gliomas is consistent with the pathophysiology of high-grade gliomas causing more inflammation, edema, and displacement of the brain due to their more aggressive nature, while low-grade gliomas are slower growing, allowing for more compensation that may explain the lower delirium risk [57]. Of note, Huang et al. (2022) hypothesized that insufficient brain compensation may be an important pathological basis for this increased risk of delirium, and this may explain the reason why higher-grade tumors place patients at a higher risk for delirium [43]. Future studies could provide further investigation into the risk of delirium for patients with brain metastases based on primary tumor types.

While this is the first reported meta-analysis studying the risk of delirium by brain tumor location, we hypothesized that tumors in areas associated with attention, such as the frontal lobes, would have higher rates of delirium. We found that patients with frontotemporal tumors had the highest risk for delirium. The frontotemporal lobes play a key role in the dorsal and ventral attention networks. Since inattention is a key feature of delirium, the tumors in the frontotemporal lobes can easily cause disruption of the networks in these areas and lead to an increased risk of delirium [58,59,60]. Furthermore, in other patient populations, dysfunction in cortical networks, such as frontoparietal networks, has been shown to be involved in the pathophysiology of delirium [9]. It is unclear whether dysregulation in these networks causes delirium or reflects underlying comorbidities, and one aim for this study was to understand the relationship between tumor location and delirium risk. Future studies should explore the underlying cortical networks and pathophysiology of delirium for brain tumor patients, with a goal of understanding the extent to which network dysregulation plays a causal role in the development of delirium.

Additionally, our meta-analysis demonstrated that the presence of delirium is correlated with an increased length of hospitalization. This is consistent with previous reports of increased lengths of hospitalizations for those with delirium [61,62,63]. These findings underscore the importance of implementing targeted delirium prevention protocols specifically tailored to the neuro-oncology population.

Finally, we showed that among brain tumor patients with delirium, patients are most likely to have hypoactive delirium, followed by hyperactive delirium, and then mixed delirium. Some of this may be due to reporting bias, given that, generally in the literature, mixed delirium is reported less frequently than hyperactive or hypoactive delirium. However, of interest is that brain tumor patients were more likely to experience hypoactive delirium than hyperactive delirium. Within the broader literature, the rates of hyperactive and hypoactive delirium in all hospitalized patients are varied, though oftentimes, hyperactive delirium is more commonly reported due to the fact that it is easier to recognize clinically [64,65,66,67]. Therefore, the fact that the rates of hypoactive delirium were higher in this population may point toward an important logical consideration of the drivers of delirium in this patient population. It is important to note, however, that given only four studies in this meta-analysis classified the different subtypes of delirium, further work should be performed to further understand the different ways delirium can present in brain tumor patients.

Our systematic review and meta-analysis used a rigorous search strategy with strict inclusion and exclusion criteria, offering a representative summary of the published literature evaluating delirium and brain tumors. Our sensitivity analyses strengthened the results by confirming their significance, despite study heterogeneity in how delirium diagnoses were made. Heterogeneity is an inherent limitation in systematic reviews and meta-analyses, especially in studies involving delirium [68,69]. In part, study heterogeneity can be due to the method of delirium assessment; studies using billing codes [32] are likely to underestimate delirium incidence, while studies using CAM-ICU [18,28,34,35,40,43,46,47,48,50] are likely to overestimate delirium incidence. Further clarification of potential sources of heterogeneity through subgroup analyses, such as brain tumor type, was unable to be performed due to limited availability in the data. For instance, our meta-analysis included both postoperative and general hospitalized patients, but we were unable to perform subanalyses based on the reason for hospitalization due to an insufficient number of studies. Furthermore, the time since diagnosis or the treatment received was not reported, both of which are important factors that we were unable to analyze due to limitations in the literature.

Many studies were also retrospective in nature, with clinical judgment being the classification method for delirium, and did not use standardized delirium measurement tools. Our bias assessment through funnel plots, Egger’s statistical test, and trim and fit tests showed high heterogeneity. The Egger’s test being statistically significant for bias likely reflects the heterogeneity of the data as well as methodological issues in smaller studies. The trim and fit test provided a possible “true” range of delirium occurrence rates of 0.04–0.14 for all brain tumors and 0.24–0.64 for gliomas and brain metastases, suggesting small-study effects or some degree of publication bias and high heterogeneity. Of note, this trim-and-fill method assumes asymmetry is purely due to publication bias, but other factors, such as study design differences or true occurrence variability, could also affect these results. Where we were able to further subdivide patients by tumor type, we found improvements in heterogeneity. Our methodology was rigorous, and the mismatch between the pooled occurrence and the trim-and-fill analysis reflects an inherent limitation in the literature and a need for high-quality research in understanding the occurrence rates of delirium in particular types of brain tumors.

However, our study is the first attempt at better understanding the overall occurrence of delirium in hospitalized brain tumor patients and reflects different delirium rates between primary and non-primary brain tumors and higher- versus lower-grade brain tumors. It also positively reflects the literature for other patients with neurologic diseases with regard to increased length of stay for patients with delirium in the hospital. Our secondary analysis also indicates an underlying pathophysiology of delirium by pointing toward a possible localization of delirium. Further research looking at neural networks and patterns of dysfunction could further help in better understanding the localization of delirium [9,70].

Additionally, when comparing the studies that measured delirium occurrence during a timeframe of greater than a week and less than a week, there were no significant differences. Theoretically, though assessing over a longer period should give a higher occurrence given a longer time for incident delirium, our data are consistent with prior studies where the majority of cases of delirium were detected on the first day of hospital admission and within five days of admission [10,71,72]. This suggests the importance of early delirium detection, and prevention strategies are crucial not only in brain tumors but in all hospitalized patients with delirium.

## 5. Conclusions

Our findings demonstrate that delirium affects 17% of hospitalized brain tumor patients overall, with significantly higher rates observed in patients with brain metastases (31%) compared to high-grade gliomas (21%) and low-grade gliomas (10%). The predominance of hypoactive delirium and the association between frontotemporal tumor location and increased delirium risk provide important insights into the pathophysiology and clinical presentation in this population. These results underscore the need for implementing targeted delirium prevention protocols and early detection strategies specifically tailored to neuro-oncology patients.

In conclusion, this meta-analysis provides the groundwork for understanding the risk of delirium in patients with brain tumors, based upon tumor type, tumor location, and setting. While all hospitalized patients have a risk of experiencing confusion and delirium, those with brain tumors may have an increased risk due to the location of the tumor. This work suggests that tumor grade and location may affect the relative risk of experiencing delirium during hospitalization. This paper contributes to the literature a better understanding of the occurrence rates and factors associated with increased delirium risk in this patient population.

## Figures and Tables

**Table 1:** Description and demographics of the included studies. Liu et al. (2023) [35] was split into two groups based on tumor type.

Author, Year	Setting	Number of Patients, *n*	Delirium Assessment Tool	Post Operative (Yes/No)	Patient Population	Delirium Prevalence (%)	Age (Mean)	Women (%)
Brauchitsch et al., 2022 [27]	Hospital	68	Clinical Judgement	N	Primary, Metastasis	13.2	59.1	-
Budėnas et al., 2018 [44]	Hospital	522	CAM-ICU	Y	Primary, Metastasis	4.2	57.23	64
Bunevicius et al., 2011 [45]	Hospital	38	DSM-IV-TR	Y	Meningioma, Glioma, Pituitary Tumors, Brain Tumors	11	-	-
Chen et al., 2020 [28]	Hospital	893	CAM-ICU	Y	Meningioma, Adenoma, Glioma	14.3	-	-
Farriols Danés et al., 2016 [29]	Palliative Care Unit	61	DSM-IV-TR	N	Primary, Metastasis	39.3	-	-
Flanigan et al., 2018 [30]	Hospital	554	Clinical Judgement	Y	Glioma	6.8	60.8	42.8
French et al., 2021 [31]	Hospital	500	DSM-V	Y	Primary, Metastasis	18.6	-	-
Fu et al., 2024 [32]	Hospital	28,340	ICD-10-CM	Y	Meningioma, Glioma, Metastasis	4.79	60.3	52.8
Gofton et al., 2012 [33]	Hospital	168	Clinical Judgement	N	Primary, Metastasis	27.4	60	42.9
Gu et al., 2022 [34]	Hospital	618	CAM-ICU	Y	Benign, Malignant	21.2	47	60.8
He et al., 2021 [46]	Hospital	29	CAM-ICU	Y	Not specified	10.3	48	50
Huang et al., 2022 [43]	Hospital	335	CAM-ICU	Y	Glioma	37.3	-	47.3
Kim et al., 2020 [18]	Hospital	969	CAM-ICU	Y	Non-functioning pituitary tumors	0.6	46.68	41.9
Kim et al., 2020 (2) [18]	Hospital	317	CAM-ICU	Y	Functioning pituitary tumors	0	44.06	53.6
Li et al., 2023 [47]	Hospital	130	CAM-ICU	Y	Glioma, Meningioma, Others (cholesteatoma, epidermoid cyst, subependymoma, and metastasis)	46.2	45	66
Liu et al., 2023 [35]	Hospital	348	CAM-ICU	Y	Pituitary Adenoma	10.34	48.3	52.2
Matsuo et al., 2017 [48]	Hospital	18	CAM-ICU	N	Metastasis	22	-	-
Ramírez-Bermúdez et al., 2010 [20]	Hospital	212	DRS-98	N	Not specified (Central Neoplasms)	55.2	-	-
Regli LKP et al., 2023 [37]	Hospital	271	Clinical Judgement	N	Glioblastoma	6.6	-	-
Sizoo et al., 2012 [38]	Hospital	101	Clinical Judgement	N	Glioma	11.8	-	28
Thisayakorn et al., 2022 [19]	Hospital	42	Clinical Judgement	N	High Grade Glioma	0	58.6	47.6
Tremont-Lukats et al., 2003 [39]	Hospital	103	Clinical Judgement	N	Metastasis	51	-	-
Wang et al., 2021 [40]	Hospital	659	CAM-ICU	Y	Benign and Malignant	18.8	48	59.8
Yamanaka et al., 2011 [41]	Palliative Care Unit	55	Clinical Judgement	N	Metastasis	19	60.7	.
Yuan et al., 2023 [49]	Hospital	150	Nu-DESC	Y	Meningioma, Glioma, Others	28.7	55	54.7
Yuan et al., 2024 [50]	Hospital	133	CAM-ICU	Y	Glioma	31.6	-	-
Zhang et al., 2024 [42]	Hospital	213	4AT	Y	Adenoma	29.58	-	-
Zipser et al., 2019 [51]	Hospital	111	Delirium Observation Screening Scale (DOS)	Y	Not specified (Cerebral Neoplasm)	29.7	-	-
Averages (%)						20	53	51

## Data Availability

The data that support the findings of this study are available from the corresponding author, John Y. Rhee, john_rhee@dfci.harvard.edu, upon reasonable request. Due to privacy, some data may not be publicly available.

## Supplementary Materials

The following supporting information can be downloaded at: https://www.mdpi.com/article/10.3390/cancers17121998/s1, S1. Sensitivity Analysis Grading Criteria; S2. Sensitivity Analysis Grading Results; S3. Database Search Criteria; S4. Forest plot showing the prevalence of delirium stratified by low risk of bias (RoB) and high RoB scores; S5. Forest plot showing the prevalence of delirium when the CAM assessment, clinician judgement, or other delirium assessment test was used; S6. Forest plot showing the prevalence of delirium stratified for studies measuring delirium for a timeframe of greater/equal than a week and less than a week; S7. Forest plot showing the prevalence of delirium stratified for studies measuring delirium at multiple timepoints and a single timepoint; S8. Bias Assessment; Table S1. PRISMA 2020 Checklist; Table S2. PRISMA 2020 for Abstracts Checklist.
