# Supplementary material for: Occurrence Rates of Delirium in Brain Tumor Patients: A Systematic Review and Meta-Analysis
Source: Cancers (Basel). 2025 Jun 15;17(12):1998. doi: 10.3390/cancers17121998 (PMC12191215; doi:10.3390/cancers17121998)
Supplement: Supplementary file 1 [file cancers-17-01998-s001.zip › cancers-3676525-supplementary.pdf]

S1. Sensitivity Analysis Grading Criteria.

The sensitivity analysis grading was adapted from Moola et al (2020).

Adapted from: Moola S, Munn Z, Tufanaru C, Aromataris E, Sears K, Sfetcu R, Currie M, Qureshi R, Mattis P, Lisy K, Mu P-F. Chapter 7: Systematic reviews of etiology and risk . In: Aromataris E, Munn Z (Editors). Moola S r*JBI Manual for Evidence Synthesis.* JBI, 2020. Available from https://synthesismanual.jbi.global

1. Were the criteria for inclusion in the sample clearly defined?

Description: The authors should provide clear inclusion and exclusion criteria that they developed prior to recruitment of the study participants. The inclusion/exclusion criteria should be specified (e.g., risk, stage of disease progression) with sufficient detail and all the necessary information critical to the study.

Yes: As close to all brain tumor patients as possible. OK if brain metastases, OK if limited to a certain age, but if too many other exclusions downgraded to "No".

2. Were the study subjects and the setting described in detail?

Description: The study sample should be described in sufficient detail so that other researchers can determine if it is comparable to the population of interest to them. The authors should provide a clear description of the population from which the study participants were selected or recruited, including demographics, location, and time period.

Yes: Clearly indicated demographics for study cohort as well as where and when study was conducted.

3. Was the exposure (Brain Tumor) measured in a valid and reliable way?

Description: The study should clearly describe the method of measurement of exposure. Assessing validity requires that a 'gold standard' is available to which the measure can be compared. The validity of exposure measurement usually relates to whether a current measure is appropriate or whether a measure of past exposure is needed. Reliability refers to the processes included in an epidemiological study to check repeatability of measurements of the exposures. These usually include intra-observer reliability and inter-observer reliability.

Yes: Clear process for diagnosing brain tumors, such as by expert analysis or imaging.

4. Were objective, standard criteria used for classification of the condition (brain tumor)?

Description: It is useful to determine if patients were included in the study based on either a specified diagnosis or definition. This is more likely to decrease the risk of bias.

Characteristics are another useful approach to matching groups, and studies that did not use specified diagnostic methods or definitions should provide evidence on matching by key characteristics.

Yes: Clear description of neuroimaging (CT or MRI), and given a "Yes" if manually or automatically outlined lesion locations or at least used a validated scale for other pathology.

5. Were confounding factors identified?

Description: Confounding has occurred where the estimated intervention exposure effect is biased by the presence of some difference between the comparison groups (apart from the exposure investigated/of interest). Typical confounders include baseline characteristics, prognostic factors, or concomitant exposures (e.g. smoking). A confounder is a difference between the comparison groups and it influences the direction of the study results. A high-quality study at the level of cohort design will identify the potential confounders and measure them (where possible). This is difficult for studies where behavioral, attitudinal or lifestyle factors may impact on the results.

Yes: Report of at least two of the following: Age, Dementia, Coma, Aphasia, Seizure, Pain, sex

6. Were strategies to deal with confounding factors stated?

Description: Strategies to deal with effects of confounding factors may be dealt within the study design or in data analysis. By matching or stratifying sampling of participants, effects of confounding factors can be adjusted for. When dealing with adjustment in data analysis, assess the statistics used in the study. Most will be some form of multivariate regression analysis to account for the confounding factors measured.

Yes: Multivariable model or stratified approach with brain tumor data included as an independent variable and delirium as the dependent variable.

7. Were the outcomes (Delirium) measured in a valid and reliable way?

Description: If for e.g. lung cancer is assessed based on existing definitions or diagnostic criteria, then the answer to this question is likely to be yes. If lung cancer is assessed using

observer reported, or self-reported scales, the risk of over- or under-reporting is increased, and objectivity is compromised. Importantly, determine if the measurement tools used were validated instruments as this has a significant impact on outcome assessment validity. Having established the objectivity of the outcome measurement (e.g. lung cancer) instrument, it's important to establish how the measurement was conducted. Were those involved in collecting data trained or educated in the use of the instrument/s? (e.g. radiographers). If there was more than one data collector, were they similar in terms of level of education, clinical or research experience, or level of responsibility in the piece of research being appraised?

Yes: Either prospective DSM based diagnosis by expert or validated clinical scale (CAM, CAM-ICU, 4AT, e.g).

8. Was appropriate statistical analysis used?

Description: As with any consideration of statistical analysis, consideration should be given to whether there was a more appropriate alternate statistical method that could have been used.

The methods section should be detailed enough for reviewers to identify which analytical techniques were used (in particular, regression or stratification) and how specific confounders were measured. For studies utilizing regression analysis, it is useful to identify if the study identified which variables were included and how they related to the outcome. If stratification was the analytical approach used, were the strata of analysis defined by the specified variables?

Additionally, it is also important to assess the appropriateness of the analytical strategy in terms of the assumptions associated with the approach as differing methods of analysis are based on differing assumptions about the data and how it will respond.

Yes: If included report of how statistical analysis was used, given "Yes" if good description with at least basic counts and group number.

S2. Sensitivity Analysis Grading Results. A lower score signifies a lower risk of bias. For each question, a value of 1 signifies low risk while a value of 0 indicates a high-risk study. Maximum overall score of 7.

S3. Database Search Criteria.

Below is the search criteria for three search engines. The search was built with the help of a librarian from the University of Navarra.

S4. Forest plot showing the prevalence of delirium stratified by low risk of bias (RoB) and high RoB scores. A random effects model in R was used with the box representing the proportion of delirium and the line representing the 95% CI.

S5. Forest plot showing the prevalence of delirium when the CAM assessment, clinician judgement, or other delirium assessment test was used. Other tests included ICD-10 review, Nursing Delirium Screening Scale, 4A's Test, and the Delirium Observation Screening Scale. A random effects model in R was used with the box representing the proportion of delirium and the line representing the 95% CI.

S6. Forest plot showing the prevalence of delirium stratified for studies measuring delirium for a timeframe of greater/equal than a week and less than a week. A random effects model in R was used with the box representing the proportion of delirium and the line representing the 95% CI.

S7. Forest plot showing the prevalence of delirium stratified for studies measuring delirium at multiple timepoints and a single timepoint. A random effects model in R was used with the box representing the proportion of delirium and the line representing the 95% CI.

S8. Bias Assessment.

Bias assessment for the meta-analysis was conducted using a funnel plot for (A) all brain tumors, and (B) after applying the Trim and Fill method, as well as (C) for gliomas and metastases only and (D) after applying the Trim and Fill method; (E) shows a funnel plot for only glioma patients.

Table S1. PRISMA 2020 Checklist

PRISMA 2020 Checklist

Section and Topic	Item #	Checklist item	Location where item is reported
**TITLE**			
Title	1	Identify the report as a systematic review.	p.1
**ABSTRACT**			
Abstract	2	See the PRISMA 2020 for Abstracts checklist.	p. 2
**INTRODUCTION**			
Rationale	3	Describe the rationale for the review in the context of existing knowledge.	p. 4
Objectives	4	Provide an explicit statement of the objective(s) or question(s) the review addresses.	p. 4
**METHODS**			
Eligibility criteria	5	Specify the inclusion and exclusion criteria for the review and how studies were grouped for the syntheses.	p. 4-5
Information sources	6	Specify all databases, registers, websites, organisations, reference lists and other sources searched or consulted to identify studies. Specify the date when each source was last searched or consulted.	p. 5
Search strategy	7	Present the full search strategies for all databases, registers and websites, including any filters and limits used.	p. 4-5, appendix 3
Selection process	8	Specify the methods used to decide whether a study met the inclusion criteria of the review, including how many reviewers screened each record and each report retrieved, whether they worked independently, and if applicable, details of automation tools used in the process.	pp. 5
Data collection process	9	Specify the methods used to collect data from reports, including how many reviewers collected data from each report, whether they worked independently, any processes for obtaining or confirming data from study investigators, and if applicable, details of automation tools used in the process.	p. 5
Data items	10a	List and define all outcomes for which data were sought. Specify whether all results that were compatible with each outcome domain in each study were sought (e.g. for all measures, time points, analyses), and if not, the methods used to decide which results to collect.	pp. 5-6, appendix 1
	10b	List and define all other variables for which data were sought (e.g. participant and intervention characteristics, funding sources). Describe any assumptions made about any missing or unclear information.	pp. 5-6, appendix 1
Study risk of bias assessment	11	Specify the methods used to assess risk of bias in the included studies, including details of the tool(s) used, how many reviewers assessed each study and whether they worked independently, and if applicable, details of automation tools used in the process.	p. 5, appendix 1
Effect measures	12	Specify for each outcome the effect measure(s) (e.g. risk ratio, mean difference) used in the synthesis or presentation of results.	pp. 6
Synthesis methods	13a	Describe the processes used to decide which studies were eligible for each synthesis (e.g. tabulating the study intervention characteristics and comparing against the planned groups for each synthesis (item #5)).	pp. 5-6, appendix 1
	13b	Describe any methods required to prepare the data for presentation or synthesis, such as handling of missing summary statistics, or data conversions.	p. 6
	13c	Describe any methods used to tabulate or visually display results of individual studies and syntheses.	p. 6
	13d	Describe any methods used to synthesize results and provide a rationale for the choice(s). If meta-analysis was performed, describe the model(s), method(s) to identify the presence and extent of statistical heterogeneity, and software package(s) used.	p. 6
	13e	Describe any methods used to explore possible causes of heterogeneity among study results (e.g. subgroup analysis, meta-regression).	p. 6
	13f	Describe any sensitivity analyses conducted to assess robustness of the synthesized results.	pp. 4-5
Reporting bias assessment	14	Describe any methods used to assess risk of bias due to missing results in a synthesis (arising from reporting biases).	p. 5-6, appendix 1
Certainty assessment	15	Describe any methods used to assess certainty (or confidence) in the body of evidence for an outcome.	p. 5-6, appendix 1

PRISMA 2020 Checklist

Section and Topic	Item #	Checklist item	Location where item is reported
**RESULTS**			
Study selection	16a	Describe the results of the search and selection process, from the number of records identified in the search to the number of studies included in the review, ideally using a flow diagram.	p. 6, figure 1
	16b	Cite studies that might appear to meet the inclusion criteria, but which were excluded, and explain why they were excluded.	p. 6
Study characteristics	17	Cite each included study and present its characteristics.	p. 6, table 1
Risk of bias in studies	18	Present assessments of risk of bias for each included study.	appendix 2
Results of individual studies	19	For all outcomes, present, for each study: (a) summary statistics for each group (where appropriate) and (b) an effect estimate and its precision (e.g. confidence/credible interval), ideally using structured tables or plots.	pp 6-7, figures 2-4
Results of syntheses	20a	For each synthesis, briefly summarise the characteristics and risk of bias among contributing studies.	figures 2-4, appendix 2,4
	20b	Present results of all statistical syntheses conducted. If meta-analysis was done, present for each the summary estimate and its precision (e.g. confidence/credible interval) and measures of statistical heterogeneity. If comparing groups, describe the direction of the effect.	pp 6-7, figures 2-4
	20c	Present results of all investigations of possible causes of heterogeneity among study results.	p. 8, appendix 4
	20d	Present results of all sensitivity analyses conducted to assess the robustness of the synthesized results.	appendix 2
Reporting biases	21	Present assessments of risk of bias due to missing results (arising from reporting biases) for each synthesis assessed.	appendix 4
Certainty of evidence	22	Present assessments of certainty (or confidence) in the body of evidence for each outcome assessed.	pp 7-8, figures 2-4
**DISCUSSION**			
Discussion	23a	Provide a general interpretation of the results in the context of other evidence.	pp. 8-9
	23b	Discuss any limitations of the evidence included in the review.	p. 10
	23c	Discuss any limitations of the review processes used.	p. 10
	23d	Discuss implications of the results for practice, policy, and future research.	pp. 9-10
**OTHER INFORMATION**			
Registration and protocol	24a	Provide registration information for the review, including register name and registration number, or state that the review was not registered.	pp. 2,6
	24b	Indicate where the review protocol can be accessed, or state that a protocol was not prepared.	pp. 2,6
	24c	Describe and explain any amendments to information provided at registration or in the protocol.	p. 6
Support	25	Describe sources of financial or non-financial support for the review, and the role of the funders or sponsors in the review.	p. 2,11
Competing interests	26	Declare any competing interests of review authors.	p. 11
Availability of data, code and other materials	27	Report which of the following are publicly available and where they can be found: template data collection forms; data extracted from included studies; data used for all analyses; analytic code; any other materials used in the review.	p. 11

From: Page MJ, McKenzie JE, Bossuyt PM, Boutron I, Hoffmann TC, Mulrow CD, et al. The PRISMA 2020 statement: an updated guideline for reporting systematic reviews. BMJ 2021;372:n71. doi: 10.1136/bmj.n71

Table S2. PRISMA 2020 for Abstracts Checklist

PRISMA 2020 for Abstracts Checklist

Section and Topic	Item #	Checklist item	Reported (Yes/No)
**TITLE**			
Title	1	Identify the report as a systematic review.	Yes
**BACKGROUND**			
Objectives	2	Provide an explicit statement of the main objective(s) or question(s) the review addresses.	Yes
**METHODS**			
Eligibility criteria	3	Specify the inclusion and exclusion criteria for the review.	
Information sources	4	Specify the information sources (e.g. databases, registers) used to identify studies and the date when each was last searched.	Yes
Risk of bias	5	Specify the methods used to assess risk of bias in the included studies.	
Synthesis of results	6	Specify the methods used to present and synthesise results.	Yes
**RESULTS**			
Included studies	7	Give the total number of included studies and participants and summarise relevant characteristics of studies.	Yes
Synthesis of results	8	Present results for main outcomes, preferably indicating the number of included studies and participants for each. If meta-analysis was done, report the summary estimate and confidence/credible interval. If comparing groups, indicate the direction of the effect (i.e. which group is favoured).	Yes
**DISCUSSION**			
Limitations of evidence	9	Provide a brief summary of the limitations of the evidence included in the review (e.g. study risk of bias, inconsistency and imprecision).	Yes
Interpretation	10	Provide a general interpretation of the results and important implications.	Yes
**OTHER**			
Funding	11	Specify the primary source of funding for the review.	Yes
Registration	12	Provide the register name and registration number.	Yes

From: Page MJ, McKenzie JE, Bossuyt PM, Boutron I, Hoffmann TC, Mulrow CD, et al. The PRISMA 2020 statement: an updated guideline for reporting systematic reviews. BMJ 2021;372:n71. doi: 10.1136/bmj.n71. This work is licensed under CC BY 4.0. To view a copy of this license, visit https://creativecommons.org/licenses/by/4.0/
